# Insect–microbe interactions and their influence on organisms and ecosystems

**DOI:** 10.1002/ece3.11699

**Published:** 2024-07-21

**Authors:** Jocelyn R. Holt, Nathalia Cavichiolli de Oliveira, Raul F. Medina, Antonino Malacrinò, Amelia R. I. Lindsey

**Affiliations:** ^1^ Department of BioSciences Rice University Houston Texas USA; ^2^ Don Bosco Catholic University Campo Grande Mato Grosso do Sul Brazil; ^3^ Department of Entomology Texas A&M University, Minnie Bell Heep Center College Station Texas USA; ^4^ Department of Agriculture Università Degli Studi Mediterranea di Reggio Calabria Reggio Calabria Italy; ^5^ Department of Entomology University of Minnesota St. Paul Minnesota USA

**Keywords:** bacteria, fungi, host–microbe interactions, microbiome, multitrophic interactions, symbiosis, virus

## Abstract

Microorganisms are important associates of insect and arthropod species. Insect‐associated microbes, including bacteria, fungi, and viruses, can drastically impact host physiology, ecology, and fitness, while many microbes still have no known role. Over the past decade, we have increased our knowledge of the taxonomic composition and functional roles of insect‐associated microbiomes and viromes. There has been a more recent shift toward examining the complexity of microbial communities, including how they vary in response to different factors (e.g., host genome, microbial strain, environment, and time), and the consequences of this variation for the host and the wider ecological community. We provide an overview of insect–microbe interactions, the variety of associated microbial functions, and the evolutionary ecology of these relationships. We explore the influence of the environment and the interactive effects of insects and their microbiomes across trophic levels. Additionally, we discuss the potential for subsequent synergistic and reciprocal impacts on the associated microbiomes, ecological interactions, and communities. Lastly, we discuss some potential avenues for the future of insect‐microbe interactions that include the modification of existing microbial symbionts as well as the construction of synthetic microbial communities.

## Graphical abstract

Insect‐symbiont interactions can have a variety of impacts on organisms and the ecosystem. These multitrophic interactions can result in differential attraction of beneficial insects or natural enemies. Additinoally, these interactions can influence plant and/or soil health as well as microbiome composition. The environment cna also modulate insect‐microbe associations with climate change projected to have consequences on these ecological interactions.

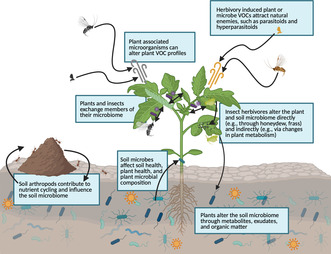

## 1 INTRODUCTION

Microbial symbionts of insects and other arthropods span a continuum from parasitism to mutualism. These include pathogenic invaders, obligate symbionts that provide essential nutrients, and a wide range of facultative associates that provide functions such as protection against natural enemies or detoxification (Ceja‐Navarro et al., 2015; Hu et al., 2018; Jahnes & Sabree, 2020; Oliver et al., 2010). Much of our knowledge about microbes associated with insects and other arthropods is based on individual symbiont species (e.g., *Buchnera aphidicola*, *Wolbachia*, and others) or the pathogens these animals transmit (e.g., *Plasmodium* sp., *Candidatus* Liberibacter asiaticus, *Borrelia burgdorferi*, *Typanosoma cruzui*, and others); see reviews on this topic (Heck, 2018; Kikuchi, 2009; Laroche et al., 2018; Moran et al., 2008; Oliver et al., 2010). While there is a great body of knowledge on microbial symbionts, exciting discoveries about the diversity of functions associated with individual symbionts are still being made in part due to tools such as high‐throughput sequencing (Almeida et al., 2017; Cheng et al., 2017, 2018; Holt et al., 2020; Muñoz‐Benavent et al., 2021; Reis et al., 2020).

Recent evidence suggests that symbiont‐mediated phenotypes are more complex and context‐dependent in nature than what has been revealed by previous studies on individual microbe species and their insect hosts. For example, in *Drosophila* spp., there are numerous lines of evidence for significant interactions between the gut microbial community, intracellular symbionts (e.g., *Wolbachia*, *Spiroplasma*), pathogenic microbes, and host physiology, which ultimately impact fly metabolism, development, immunity, and even behavior (Chen et al., 2019; Delbare et al., 2020; Fischer et al., 2017; Fromont et al., 2019; Lee & Brey, 2013; Simhadri et al., 2017; Ye et al., 2017). The composition of the gut microbiome itself is a result of numerous factors, including competition between microbes, the physical and chemical properties of the insect gut, and stochastic processes (Flynn et al., 2021; Itoh et al., 2018). Such complex species interactions can result in significant variability in the fitness and phenotype of the host, which can ultimately cascade to entire insect populations and associated communities (Ferrari & Vavre, 2011; Frago et al., 2012; Leclair et al., 2017; McLean, 2019; McLean et al., 2017). For example, plants can respond to aphid herbivory by altering their volatile organic compound (VOC) profile to attract aphid natural enemies. However, if aphids host the facultative symbiont *Hamiltonella defensa* the VOC profile is altered in a way that instead reduces the plant's attractiveness toward the aphids' natural enemies (Frago et al., 2012). Aphids hosting *H. defensa* are also reported to cause reductions in aboveground plant growth (Bennett et al., 2016), which then impacts a suite of other ecological interactions.

Insect‐associated microbes can have complex interactions and we discuss how symbioses can change across communities and environments. In this review, we explore the influence of the environment on insect–microbe interactions and the downstream consequences on host evolutionary ecology. We discuss applied perspectives for a deeper understanding of insect–microbe interactions, and how their manipulation can help us to solve agricultural and environmental challenges. We conclude with considerations for continued research of insect–microbe–environment interactions and its applications.

## 2 THE DIVERSITY OF MICROBIAL IMPACTS ON INSECTS

### 2.1 Functions of insect symbionts

The functional contributions of microbes to insect biology are diverse, and symbionts are often classified according to how they impact host biology (i.e., nutritional, defensive, or reproductive symbionts). However, it is becoming increasingly clear that many of these roles or emergent phenotypes depend on a wide range of biological, ecological, and environmental contexts, and are often just one of the many phenotypes that a symbiont might be responsible for. Symbionts that provide a suite of context‐dependent phenotypes are perhaps more common than has been realized, and there are likely many yet to be discovered impacts associated with even the most well‐studied symbioses.

Insects that live on nutritionally poor or imbalanced diets regularly form obligate associations with microbes that synthesize these nutrients. Sap‐feeding hemipteran insects typically have 1–2 vertically transmitted symbionts that supplement the diet of the insect host with essential amino acids (Moran et al., 2008) and vitamins (Manzano‐Marın et al., 2020). Exclusively, hematophagous insects and arthropods (e.g., tsetse, bed bugs, ticks, and others) require a source of B vitamins and harbor symbionts that fulfill this need (Bonnet et al., 2017; Hosokawa et al., 2009; Michalkova et al., 2014). The diversity and mechanisms of these obligate nutritional symbioses have been explored in more focused reviews (Douglas, 2009; Rio et al., 2016; Skidmore & Hansen, 2017). In addition to obligate nutritional symbionts, the insect gut microbiota plays an important role in digestion, nutrition, and detoxification of harmful compounds in the diet (Douglas, 2015, 2018; Engel & Moran, 2013; Itoh et al., 2018; Jing et al., 2020; Mason, 2020; Muñoz‐Benavent et al., 2021; Strand, 2018). For example, bark beetles harbor *Pseudomonas* species that aid in the digestion of plant tissues (Saati‐Santamaría et al., 2021) and fungi that aid in wood decomposition preingestion (Six, 2012). Similarly, termites have a diverse gut microbiome that enables the breakdown of cellulose and lignin (Arora et al., 2022; Brune & Friedrich, 2000; Marynowska et al., 2023; Xie et al., 2023) as do some ants and bark beetles (Barcoto et al., 2020). Several insect species have gut microbiota that facilitate nitrogen recycling (Nishimura et al., 2020; Russell et al., 2009; Sabree et al., 2009). Resident gut microbiota can also facilitate the detoxification of plant defensive compounds. For example, coffee berry borers (*Hypothenemus hampei* Ferrari), flea beetles (*Psylliodes chrysocephala* L.), and ambrosia beetles harbor gut‐associated microbes that can detoxify caffeine, isothiocyanates, and other secondary metabolites, respectively (Barcoto et al., 2020; Ceja‐Navarro et al., 2015; Shukla & Beran, 2020; van den Bosch & Welte, 2017). Other gut symbionts (e.g., *Burkholderia*, *Pseudomonas*, *Citrobacter*) can confer protection against insecticides (Boush & Matsumura, 1967; Cheng et al., 2017; Kikuchi et al., 2012; Sato et al., 2021) or plant‐specialized metabolites (Ceja‐Navarro et al., 2015; Shukla & Beran, 2020; van den Bosch & Welte, 2017) through detoxification (Cheng et al., 2017; Wang et al., 2023).

In addition to supporting host nutrition and digestion, microbes can alter insect reproductive processes, such as oogenesis, sperm‐egg compatibilities, or offspring sex ratios (Kaur et al., 2021; Ma et al., 2014; Perlmutter & Bordenstein, 2020). The most common reproductive symbiont associated with these reproductive phenotypes is *Wolbachia*, a clade of Alphaproteobacteria nested within the Rickettsiales that infects approximately one‐third of all arthropods (Kaur et al., 2021; Werren et al., 2008). Other reproductive symbionts include *Cardinium*, *Spiroplasma*, *Arsenophonus*, and other nonpathogenic *Rickettsia* species (Duron, 2019; Duron & Hurst, 2013; Massey & Newton, 2022). Although symbionts that manipulate reproduction are often present in insect populations, there are instances where, despite high infection frequencies, there are no discernible reproductive manipulations, providing opportunities to discover other roles these symbionts may play in insect physiology and evolution (Hamm et al., 2014; Kriesner & Hoffmann, 2018).

Finally, there is growing recognition for the role of viruses as key insect symbionts. Viruses can confer phenotypes similar to those of the aforementioned bacterial or fungal associations. Although many viruses are categorized as pathogenic, the discoveries of beneficial viruses continue to grow (Roossinck, 2011). For example, the *Helicoverpa armigera* densovirus (HaDNV1) provides *H. armigera* with enhanced growth, fecundity, and resistance to insecticidal Bt proteins (Xu et al., 2014). A few insect‐associated viruses are responsible for male‐biased sex ratios, such as a nyamivirus in the parasitoid wasp *Pteromalus puparum*, and tomato spotted wilt orthotospovirus in the western flower thrips *Frankliniella occidentalis* (Tao et al., 2022; Wang et al., 2017). There is a wide diversity of insect viruses that can impact their host, some of which leave behind endogenous viral elements in the host's genome (Gilbert & Belliardo, 2022). While some studies have revealed how such viruses affect insect biology such as bacteriophage presence conferring the protective phenotype of *Hamiltonella defensa* (Lynn‐Bell et al., 2019), this area remains rich for discovery.

### 2.2 Symbionts influence behavior

While we often think of the metabolic and defensive functions that microbial symbionts provide, microbial symbionts can also modulate host behavior. Various mechanisms can drive this, such as symbionts releasing VOCs that increase aggregation behavior or facilitate mate attraction in insects such as cockroaches, bark beetles, and flies. In cockroaches, the microbial gut community produces volatile carboxylic acids in the feces, which drives aggregation behavior (Wada‐Katsumata et al., 2015). Other symbionts release VOCs that enhance mate attraction or act as colony recognition signals for social insects (Calcagnile et al., 2019; Chaudhury et al., 2010). Knowledge of aggregation or attraction behavior due to insect pheromones and microbial VOCs can be harnessed and applied in pest control practices to eliminate pests (Morrison 3rd et al., 2021) or to attract natural enemies (Goelen et al., 2020).

Crops and their associated microbes can similarly mediate insect behaviors and even enhance biological control efforts by attracting natural enemies. For example, pea aphids (*Acyrthosiphon pisum* Harris) avoid leaf surfaces when *Pseudomonas syringae*, a plant epiphyte or pathogen depending on the strain, is present on the surface (Hendry et al., 2018; Smee, Real‐Ramirez, et al., 2021). Cover crops and their associated microbes can similarly mediate insect behaviors and enhance biological control efforts through attracting natural enemies. Buckwheat plants (*Fagopyrum esculentum* Moench) harbor certain bacteria (i.e., *Staphylococcus epidermidis*, *Terrabacillus saccharophilus*, *Pantoea*, and *Curtobacterium*) in their floral nectar that emit microbial VOCs (Cusumano et al., 2023). These microbial VOCs attract parasitoid wasps (*Trissolcus basalis* Wollaston) to nectar (Cusumano et al., 2023), a resource that can enhance wasp fecundity and lead to population reductions in the wasp's host, the southern green stinkbug (*Nezara viridula* L.).

Additionally, there have been cases where host behaviors have been selected upon to facilitate the acquisition of microbial symbionts (Hosokawa & Fukatsu, 2020; Lanan et al., 2016). It has also been suggested that microorganisms may accelerate the evolution of complex behaviors, such as parental care since tending to offspring would facilitate the transmission of the microbiome (Gurevich et al., 2020). There are many exciting opportunities for understanding the roles that microbial symbionts may play in insect behavior, and we anticipate that there are numerous host behaviors in which microbes play a role.

### 2.3 Symbiont multifunctionality

While many symbionts provide key contributions to insect biology (Brownlie & Johnson, 2009; Kaltenpoth & Engl, 2016; Oliver & Martinez, 2014; Oliver & Perlman, 2020; Van Arnam et al., 2018), there is increasing evidence that individual symbionts can provide multiple functions to their host. Interestingly, this includes obligate symbionts well known for critical metabolic roles. For instance, *Wigglesworthia*, the obligate bacterial mutualist of tsetse, blood‐sucking flies in the genus *Glossina* (Wiedemann), provides a range of benefits to the host, including blood meal digestion and immune system function, in addition to its essential role in synthesizing and provisioning B vitamins (Bing et al., 2017; Pais et al., 2008; Weiss et al., 2011). Psyllids house two obligate symbionts *Candidatus* Carsonella ruddii and *Candidatus* Profftella armatura, the latter of which produces essential metabolites as well as the toxin diaphorin, which may protect the host from natural enemies and pathogens (Nakabachi et al., 2020). Toxin production by this symbiont may further stabilize the relationship between the psyllid and its symbiont.

*Wolbachia* is a well‐documented example of symbiont multifunctionality. This bacterial symbiont is associated with a suite of changes in host physiology that extend beyond reproduction. For example, some strains of *Wolbachia* also protect the insect host against secondary viral infections, play a role in oogenesis, and/or act as nutritional mutualists (Dedeine et al., 2001; Hosokawa et al., 2009; Ju et al., 2020; Lindsey et al., 2023; Martinez et al., 2014; Moriyama et al., 2015; Newton & Rice, 2020; Teixeira et al., 2008). Across the diversity of host‐*Wolbachia* associations, many *Wolbachia* infections do not appear to induce any “selfish” reproductive phenotype (e.g., cytoplasmic incompatibility, feminization) (Kriesner & Hoffmann, 2018). In other instances, some *Wolbachia* species can induce multiple reproductive phenotypes (e.g., male‐killing and cytoplasmic incompatibility) (Richardson et al., 2016; Sasaki et al., 2005). Indeed, the identification of additional *Wolbachia* phenotypes, reproductive and other, is changing how we think about the costs and benefits of the host‐*Wolbachia* relationship (Zug & Hammerstein, 2015).

Similarly, other facultative symbionts can have a variety of functions within or across hosts. For instance, *Bombella apis*, a bacterial associate of honey bees, protects the hive against fungal infections and synthesizes essential amino acids for the brood including lysine, an amino acid that supports development under nutrient‐poor conditions (Miller et al., 2021; Parish et al., 2022). Several *Spiroplasma* strains infecting pea aphids are capable of protecting the insect against both parasitoid wasps and fungal pathogens (McLean et al., 2020). Symbionts can have different functions in different host species. For instance, *H. defensa* can either skew sex ratios or provide a defensive benefit depending on whether it is harbored by white flies or aphids (Oliver & Higashi, 2018; Shan et al., 2019).

Many symbionts previously associated with a particular role or function can likely play multiple roles in their host. Moreover, even symbionts that are not classified as nutritional mutualists are likely to affect host metabolism due to their close association with the host gut lumen or cell cytoplasm and the associated energetic costs for the host. Many of these phenotypes have been overlooked or may be highly dependent on context, such as host genotype, environmental conditions, or the presence of other microbial symbionts. Indeed, these yet to be discovered phenotypes likely contribute to the difficulties associated with estimating their impacts on host fitness and symbiont abundance in nature (Bockoven et al., 2019; Brown et al., 2020).

## 3 EVOLUTIONARY ECOLOGY OF SYMBIONTS AND THEIR INSECT HOSTS

Across insect evolution, there is mixed support for phylosymbiosis: the co‐diversification of host phylogeny and microbial community composition. In select insect genera there is evidence of phylosymbiosis (i.e., *Nasonia*, *Drosophila*, *Formica*, *Culex*, *Aedes*, and *Anopheles*) (Brooks et al., 2016; Díaz‐Sánchez et al., 2019; Jackson et al., 2023). In other taxa there is no support for phylosymbiosis, including most ants, solitary bees, caterpillars, dragonflies, stick insects, and some species of beetles (Hammer et al., 2019); this pattern is also seen across several insect orders (Malacrinò, 2022). For some arthropod taxonomic groups, such as cockroaches (Blattodea), the detection of phylosymbiosis is stronger at shorter evolutionary times for particular microbial species, and this signal becomes weaker and microbial associations are more homogenized over longer evolutionary scales (Tinker & Ottesen, 2021). Regardless of whether microbial communities and their hosts exhibit phylosymbiosis, these microbial players are potent drivers of insect evolution (Perlmutter & Bordenstein, 2020; Zilber‐Rosenberg & Rosenberg, 2008). In many instances, the composition of microbial communities can be better explained by environmental acquisition, habitat filtering, or by neutral and stochastic processes (Sieber et al., 2019).

The mode of symbiont acquisition by insects and other arthropods can be highly variable. The impacts of acquisition and transmission on the evolution of the symbiotic partnership are well documented for obligate nutritional symbioses. Symbioses early in the evolutionary history of insects likely facilitated the transition of the host insect to a highly imbalanced diet. For instance, the microbial symbionts (i.e., *Actinobacteria*, *Clostridium*, *Klebsiella*) of red bugs (Pyrrhocoridae) are thought to have facilitated insect feeding (Sudakaran et al., 2015), despite the presence of plant defensive compounds. In an established nutritional symbiosis, those symbionts are vertically transmitted from insect parent to offspring and often experience the effects of Muller's ratchet, which leads to highly reduced bacterial genomes and extreme co‐dependence on the host (McCutcheon & Moran, 2011; Moran et al., 2008). While there are some highly conserved relationships between arthropods and microbial symbionts associated with vertical transmission (obligate or facultative) to their arthropod hosts (e.g., aphids and *B. aphidicola*, tsetse and *W. glossinidia*), there are other instances where arthropods acquire beneficial symbionts each generation. Symbionts that are acquired de novo from the environment each generation can provide the host with another avenue toward novel functions that facilitate adaptation and response to new challenges on more immediate time scales. For example, many insects form symbioses with bacteria in the family Burkholderiaceae (including the genera *Caballeronia* and *Burkholderia*), which are acquired from the environment each generation (Olivier‐Espejel et al., 2011; Takeshita & Kikuchi, 2017). In the bean bug (*Riptortus pedestris*), the availability of *Burkholderia* species in the soil enables these insects to acquire a symbiont that is essential for food processing (Kikuchi et al., 2007). Different strains and species within Burkholderiaceae can provide nutritional and/or defensive benefits to hosts; therefore, environmental acquisition offers a pool of genetic and functional diversity, facilitating the acquisition of multifunctional symbionts and adaptation to specific local conditions (Kaltenpoth & Flórez, 2020; Tago et al., 2015). For example, bean bug and stinkbug species can acquire different *Burkholderia* strains/species from the environment sometimes harboring multiple species simultaneously (Kikuchi et al., 2011), which could enable rapid shifts in the composition of bacterial species across generations. Interestingly, at least 42 different *Burkholderia* species are associated with bean bugs (Ohbayashi et al., 2020). In addition to improving bean bug growth and health, some *Burkholderia* symbionts can degrade fenitrothion‐based insecticides (Kikuchi et al., 2012), facilitating the insect host's survival toward pesticide‐based management tactics in agroecosystems.

Although the acquisition of microbial organisms can frequently occur from the environment (Agarwal et al., 2017), some insects, such as turtle ants and bean bugs, actively filter microbial symbionts from the environment. In order to filter *Burkholderia* from surrounding microorganisms in the environment, a bean bug species (*R. pedestris*) contains an area in its midgut, termed the constricted region, that is composed of microvilli and a mucus‐like matrix that can only be crossed by the symbiont *Burkholderia* (Ohbayashi et al., 2015, 2019; Takeshita & Kikuchi, 2017). The constricted region subsequently closes after acquiring *Burkholderia* species (Kikuchi et al., 2020). Turtle ants share a core microbiome (Flynn et al., 2021; Hu et al., 2018; Ramalho & Moreau, 2023) that is maintained by the development of a proventricular filter (Flynn et al., 2021; Lanan et al., 2016). Therefore, these filters ensure high fidelity for the insect host and the environmentally acquired symbiont.

For other insects, the microbiome composition as a whole is significantly associated with different environmental factors. For instance, monarch butterflies (*Danaus plexippus* L.) harbor microbial communities that are similar to the plant rhizosphere of the milkweed (*Asclepias* L.) species that the caterpillars consumed (Hansen & Enders, 2022). Similar results have been obtained in other model systems (Hannula et al., 2019; Malacrino et al., 2021; Malacrinò & Bennett, 2024) where it has also been observed that variation in the soil microbiome alters the herbivore‐associated microbial communities, in turn negatively influencing herbivore fitness (Malacrinò & Bennett, 2024). These results support the idea that steering the soil microbiome might be a viable strategy for sustainable pest management (French et al., 2021; Pineda et al., 2017). Mosquitoes are another insect that are reported to harbor different microbial communities depending on their life stage, and it has been proposed that for some mosquito species, rather than sharing a taxonomically similar microbiome, these insects share a functionally similar microbial community (Guégan et al., 2018). Importantly, invasive species may be prone to acquiring new microbial associates from the environment (Rassati et al., 2019), facilitating range expansion and their ability to inhabit new niches (Gupta & Nair, 2020). Several invasive insects are associated with symbiont switches or the novel acquisition of a symbiont community that differs from those in the native range (Li et al., 2022; Rassati et al., 2019; Taerum et al., 2013; Wingfield et al., 2016). This suggests that flexibility with new microbial associations might be a key mechanism for successful biological invasion.

Microbial symbionts can also alter the host population structure. Maternally transmitted symbionts, including *Wolbachia* and *Spiroplasma*, are well‐documented in driving genetic sweeps associated with an advantageous symbiont‐infected matriline. Several well‐characterized sweeps have occurred due to these fitness‐enhancing intracellular infections. This includes the spread of cytoplasmic incompatibility‐inducing *Wolbachia* in *Drosophila* across California in only 3 years (Turelli & Hoffmann, 1991), an anti‐nematode *Spiroplasma* proliferating in fungus consuming flies (*Drosophila neotestacea* Grimaldi, James, and Jaenike) across North America (Jaenike et al., 2010), and *Rickettsia* sweeping through whitefly populations (Himler et al., 2011). The spread of symbiont‐infected matriline results in concomitant mitochondrial sweeps, the potential spread of other microbes associated with those matrilines (e.g., provisioned gut microbes and co‐infecting vertically transmitted microbes), and changes in population genetic diversity and structure (Cariou et al., 2017; Deng et al., 2021; Graham & Wilson, 2012; Jiggins, 2003). Such sweeps are likely more pronounced for systems where reproductive symbionts (*Wolbachia*, *Cardinium*, *Rickettsia*) induce asexual parthenogenesis in their respective hosts (e.g., parasitoid wasps, thrips, and mites) (Fricke & Lindsey, 2024; Hurst et al., 2002; Kumm & Moritz, 2008; Ma & Schwander, 2017).

The transition to asexual reproduction, via symbiosis, genetic factors (Neiman et al., 2014), or environmental factors (Simon et al., 2010), has the potential to impact insect evolution in several ways. For example, asexual reproduction is associated with significant population genetic changes, including altered rates of polymorphism and mutation, differences in effective population size, higher linkage disequilibrium, more interference selection, and increased intragenomic conflict (Burke & Bonduriansky, 2017; Jaquiéry et al., 2018; Keightley & Otto, 2006; Orive, 1993; Otto, 2009; Tooby, 1982). These changes, along with the reproductive potential of a single isolated female, can result in increased invasion potential and altered adaptation dynamics to chemicals, pesticides, parasites, or changing environments (Guzmán et al., 2012; Lombardo & Elkinton, 2017; McDonald et al., 2016; Peccoud et al., 2008). Many agricultural pests, including aphids, whiteflies, and thrips, are parthenogenetic, a reproductive system associated with rapid population growth rates (Garnas et al., 2016; Hoffmann et al., 2008; Lin et al., 2017; Lombardo & Elkinton, 2017). Symbiont‐mediated parthenogenesis offers a relatively rapid switch to asexual reproduction, likely accelerating these processes (Huigens et al., 2000). Whether symbiont‐enabled or not, parthenogenesis, has the potential to facilitate the spread of invasive species or well‐adapted clones. Indeed, many insect invasions are associated with the establishment of clones, sometimes across multiple locations, in which each clone is well suited to the local environment (Hoffmann et al., 2008; Peccoud et al., 2008).

Based on the examples mentioned above, there can be interplay between insect genotypes, symbiont‐mediated genetic sweeps, and/or the environmental acquisition of symbionts that confer locally advantageous phenotypes. Indeed, the establishment of particular host genotypes may be associated with the ability to better leverage local symbiont pools (Garnas et al., 2016) or symbiont species/strains. While mutations play an important role in the evolution of organisms and their symbiotic interactions, newly acquired symbionts have the potential to facilitate rapid adaptation, niche expansion, and genetic sweeps. As new insect genotypes establish in new environments, this provides opportunities for novel interactions, thus further enabling novel host‐symbiont combinations.

## 4 ECOSYSTEM INTERACTIONS

### 4.1 Synergistic insect–microbe interactions

In addition to the potential for individual microbes to affect their hosts in multiple ways, interactions within the insect‐associated microbial community can drive emergent phenotypes of the symbiont or host (McLean et al., 2018; Weldon et al., 2020). For example, insect‐associated microbial communities can influence their host's relationship with pathogens (e.g., increased resistance) (Gerardo et al., 2020; McLaren & Callahan, 2020) that they harbor or vector, likely because resident microorganisms outcompete the pathogens, or prime the insect's immune system (Prigot‐Maurice et al., 2022). In squash bugs the establishment of the gut symbiont, *Caballeronia*, competitively excludes the plant pathogen *Serratia marcescens*, preventing transmission to the plant host (Mendiola et al., 2022). Although reported as pathogenic in other insect species (Pineda‐Castellanos et al., 2015; Sezen et al., 2001) *S. marcescens* is not currently known to be harmful to squash bugs (Heppler, 2007). The citrus pathogen *Candidatus* Liberibacter asiaticus (Huanglongbing) impacts the psyllid microbiome composition (Jiang et al., 2023). It has been suggested in Asian citrus psyllid (*Diaphorina citri Kuwayama*) that upregulation of the diaphorin toxin by one of its bacterial symbionts (*Profftella*) may reduce the insect's pathogen transmission (Ramsey et al., 2015) to host plants in the surrounding ecosystem. Genotype‐by‐genotype interactions between the symbionts *H. defensa* and *Fukatsuia symbiotica* are significant drivers of pea aphid phenotypes across a range of biotic and abiotic stressors (Smee, Raines, et al., 2021). Additionally, the bacteriophage variant (APSE or *A. pisum* secondary endosymbiont) present in *Hamiltonella* is one factor responsible for the level of resistance aphids have against parasitoids, as well as the aphid genotype, and the wasp genotype (Oliver & Higashi, 2018). Interactions between the arthropod host and its symbiont, as well as the multitrophic impacts of these associations, can alter the plasticity of the insect to accommodate novel challenges, thus facilitating adaptation to a new environment (Kolodny & Schulenburg, 2020).

**FIGURE 1:**
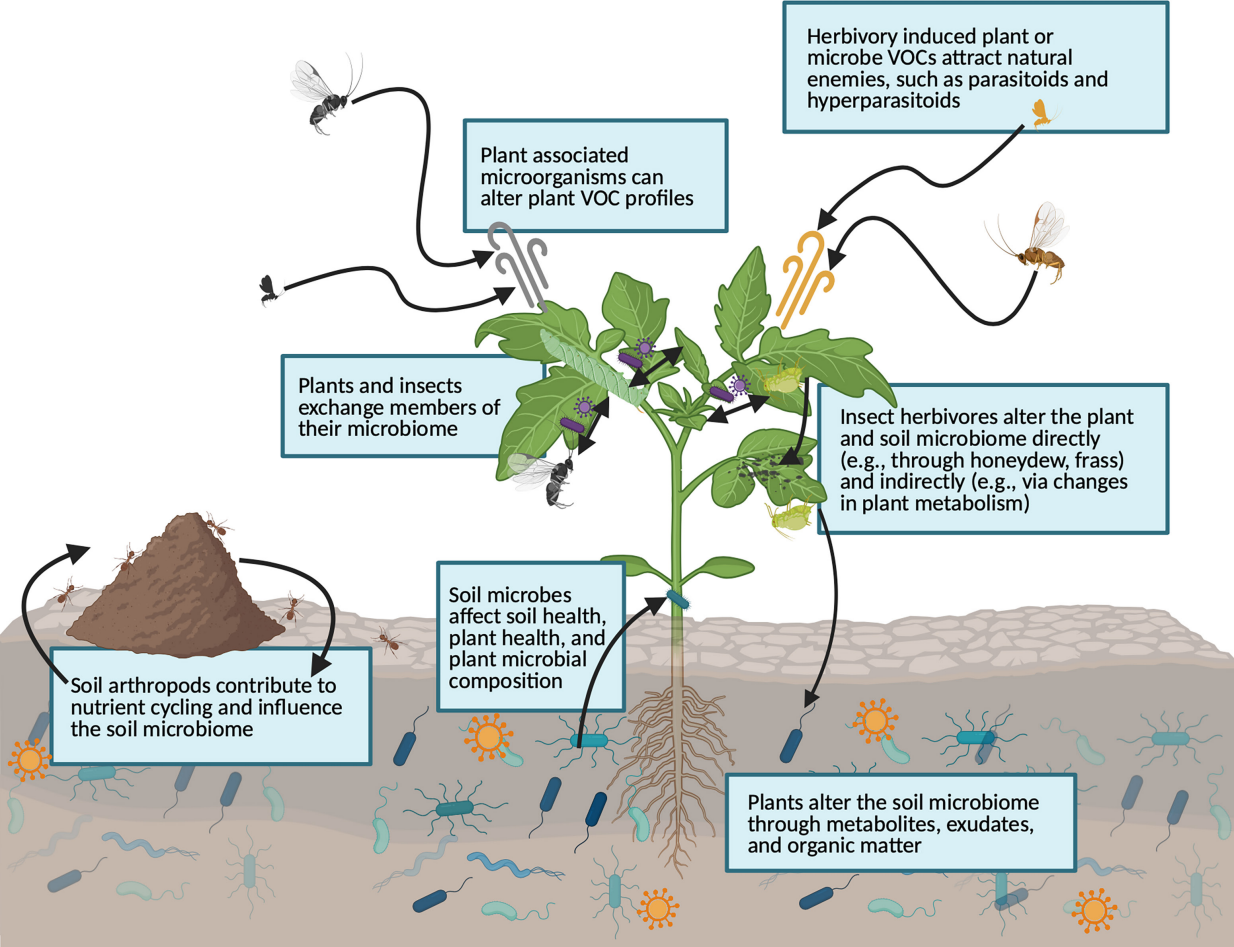
Plant–insect–microbe interactions influence the organisms and the surrounding ecosystem. Soil microbes can affect plant health and plant microbial composition. Plants and insects can exchange microbes, insects can influence plant health, and herbivore attack can trigger the release of volatile organic compounds (VOCs) from the plant or its associated microbes. Additionally, soil arthropods can move and enhance the composition of microbial organisms in the soil. Created with BioRender.com.

Additionally, insect–microbe interactions and their emergent phenotypes can affect both the host populations and surrounding communities (Ferrari & Vavre, 2011; Frago et al., 2012; Hopkins et al., 2017; Leclair et al., 2017). This seems to be a common feature of defensive symbioses (Hopkins et al., 2017). For instance, black bean aphids (*Aphis fabae* Scopoli), which harbor *H. defensa* were differentially protected from different parasitoid wasp species, resulting in shifts in the parasitoid community (Rothacher et al., 2016). These differences in herbivore and parasitoid wasp communities could differentially attract hyperparasitoids (Poelman et al., 2012) and cascade to the species composition and abundance of hyperparasitoids. Similar community level effects are seen when aphid‐secreted honeydew alters the surrounding soil microbial community (Milcu et al., 2015; Tun et al., 2020), and subsequently affects soil nutrients and plant health (Grier & Vogt, 1990; Potthast et al., 2022). Changes in the soil microbial community and nutrient availability can affect plant nutrition, health, and/or microbial associations which can then cascade to directly affect the arthropod communities associated with these plants or the contributions of arthropods to the surrounding microbial community (Bennett et al., 2006, 2016; Pineda et al., 2012; Wilkinson et al., 2019). Alterations in the soil microbial community, plant health, and organism interactions can also occur with the input of other insect by‐products such as exoskeletons and frass (Barragán Fonseca et al., 2023; van de Zande et al., 2024; Wantulla et al., 2023). These interactions can be visualized as feedback loops where environmental microbes affect other living organisms, such as insects and plants, which in turn transport or share microbial organisms back to the surrounding environment (Figure 1).

Noninsect arthropods such as ticks in the order Ixodida are also infected with a wide diversity of both pathogenic and nonpathogenic microbes, including intracellular bacteria, which together impact tick biology as well as pathogen establishment and transmission (Abraham et al., 2017; Bonnet et al., 2017; Buysse et al., 2021; Cull et al., 2021; Kitsou et al., 2021; Maitre et al., 2022). For instance, sheep ticks (*Ixodes ricinus* L.) harbored co‐infections with *B. burgdorferi* and other animal pathogens (i.e., *Lariskella*, *Rickettsia*, *Rickettsiella*, *and Spiroplasma*), a composition that can be shaped by the host tick genotype, elevational cline or geographic location, and potentially through ecological facilitation via other microbes (Aivelo et al., 2019). Furthermore, the tick life stage, elevational gradient, and location from which it was collected were also found to affect the microbiota composition of *I. ricinus* (Aivelo et al., 2019). In another instance, the presence of *Anaplasma phagocytophilum* was found to alter tick gene expression, likely facilitating host infection (Abraham et al., 2017). These interactive effects could increase pathogen transmission, alter vector and disease ecology, and negatively affect animal health. When considering the presence and diversity of arthropod symbionts, we should also consider how these associations may alter organism interactions and ecosystem functions.

### 4.2 Insect‐associated viruses as drivers of multi‐trophic interactions

There is a growing understanding of the role of insect‐associated viruses in mediating interspecific interactions, particularly between insects and their plant or animal hosts. This has been well documented for insect‐transmitted phytopathogenic viruses. For example, infection with tomato yellow leaf curl virus (*Begomovirus* spp.) promotes enhanced fecundity and longevity in its whitefly (*Bemisia tabaci* Gennadius) vector (Jiu et al., 2007). The association of whiteflies with *Begomovirus* is correlated with the presence of additional microbial symbionts (Gottlieb et al., 2010; Gueguen et al., 2017; Su et al., 2013). Interestingly, the positive effect on whiteflies was not a result of infection within a given whitefly, but rather, a consequence of feeding on virus‐infected plants (Jiu et al., 2007). Similarly, increased population growth of whiteflies was also reported for infection with a tomato yellow leaf curl virus strain isolated from Israel (Maluta et al., 2014). The tomato whitefly chlorosis virus (ToCV) (Huang et al., 2021) increased the fecundity of *B. tabaci* through elevated expression of vitellogenin (Vgt). This example provides an underlying molecular mechanism for vector fecundity modulated by the phytopathogenic virus to facilitate viral dissemination (Huang et al., 2021).

Persistent plant viruses, such as pepper cryptic virus 1 (PCV‐1), are hypothesized to manipulate host plant odors, reducing plant attractiveness to aphids (*Myzus persicae* Sulzer) that vector pathogenic viruses (Safari et al., 2019). Considering that most pepper plants (*Capsicum annuum* L.) are associated with these persistent viruses, the beneficial role of PCV‐1 in repelling insect herbivores could be the result of a much longer evolutionary history between pepper cryptic viruses and their pepper host (Safari et al., 2019). Additionally, the defensive function of PCV‐1 against aphids could cascade to surrounding uninfected plants by either deterring aphids from the wider region or driving the aphids toward alternate host plants.

Other nonphytopathogenic insect‐associated viruses affect a wide range of insect traits, including pathogen resistance and adaptation to new plant hosts. For example, the *Acyrthosiphon pisum* virus (APV) has enabled pea aphids to adapt to two new plant hosts that were previously unfavorable for aphid development. Mutations in APV RNA‐dependent RNA polymerase promote increased APV replication, leading to the suppression of plant defenses against pea aphids (Lu et al., 2022). Specifically, the APV reduces the plant levels of the phytohormones jasmonic acid (JA) and JA‐isoleucine, resulting in higher aphid survival (Lu et al., 2020).

Parasitic insects, especially parasitoid wasps in the Ichneumonoidea superfamily, have obligate associations with endogenous viruses that facilitate parasitization success. Ichneumonid and Braconid wasp genomes contain endogenous polydnaviruses (PDVs) which are specifically expressed in the venom glands to generate PDV particles that are injected into a host, ultimately manipulating the host immune system to facilitate wasp development (Drezen et al., 2017; Herniou et al., 2013). In the parasitic wasp *Venturia canescens*, a functional RNA polymerase from the endogenous PDV (a nudivirus) facilitates the production of virus‐like particles that are packaged with virulence factors, thereby conferring parasitic success (Cerqueira de Araujo et al., 2022). The impacts of some PDVs extend to additional trophic levels and play prominent roles in shaping plant–herbivore interactions. For example, *Microplitis croceipes*‐associated PDVs not only downregulate the immune system of the host corn earworm (*Helicoverpa zea* Boddie) caterpillar (Tan et al., 2018) but also impact caterpillar salivary enzymes, which leads to the suppression of plant defenses. Consequently, parasitized caterpillars develop faster, favoring the survival of parasitoid wasp offspring (Tan et al., 2018). Additionally, recent studies have suggested an interaction between PDV‐associated parasitoids and the host gut microbiome. Parasitized caterpillars experienced altered gut microbiome composition and metabolite production, ultimately changing their physiology (Cavichiolli de Oliveira & Cônsoli, 2020; Zhang et al., 2022).

Given the large ecosystem impact of insect‐associated viruses, understanding the breadth and mechanisms of these interactions will contribute to the development of innovative management strategies. Furthermore, a wider knowledge base in this area may also facilitate monitoring efforts for predicting the real‐time emergence of new pathogens or other critical changes in ecological communities.

### 4.3 Impacts of environmental, plant, and soil microbiota

In addition to the effects of insect–microbe associations on ecosystems, reciprocal interactions driven by environmental microbes can alter insect biology and insect microbiomes. The microbial composition of soil and plant surfaces has been shown to play a role in shaping the presence and behavior of numerous arthropods. For example, the presence of arbuscular mycorrhizal fungi in different *Solanum* species leads to the increased emergence of parasitoid wasps (*Aphidius ervi*) that attack potato aphids (*Macrosiphum euphorbiae* Thomas) (Bennett et al., 2016).

Below ground, numerous interactions in the soil can also drive ecological changes. These include both the microbial community of the soil and soil‐dwelling insects and arthropods. Soil microbial communities are well described for driving nutrient cycling and resource acquisition by plants, which in turn affects interactions with higher trophic levels (Barea et al., 2005; Garcia & Kao‐Kniffin, 2018). Importantly, soil arthropods can change the soil microbiome. For example, soil arthropods contribute to litter decomposition by increasing microbial activity and abundance (Culliney, 2013; Hou et al., 2024; Li et al., 2021; Soong et al., 2016). The presence of soil arthropods can lead to increased microbial respiration and concentrations of nutrients such as nitrate, ammonium, and phosphate (Gergócs et al., 2022; Sagi & Hawlena, 2021; Teuben & Roelofsma, 1990). Arthropods such as springtails (Collembola) can affect nitrogen cycling, nitrogen uptake by plants, and microbial abundance, although this varies substantially across cropping systems and fertilization treatments (Gergócs et al., 2022). Decomposition processes by soil microbes and arthropods are important for soil health. In instances with lower microbial abundances, decomposition rates are reduced and can result in nitrogen‐limited habitats (Soong et al., 2016).

The soil microbial community can also be manipulated to suppress insect pests (Bennett et al., 2006; Hokkanen & Menzler‐Hokkanen, 2018; Menzler‐Hokkanen et al., 2022; Pineda et al., 2010, 2017). For example, the use of biostimulants, which are substances that promote the growth of beneficial microbes or contain beneficial microbes, can promote plant growth and health (Rouphael & Colla, 2020). In rice fields inoculated both with arbuscular mycorrhizal fungi and an insecticidal seed treatment there was reduced plant injury by rice water weevils (*Lissorhoptrus oryzophilus* Kuschel) (Bernaola & Stout, 2021). Similarly, tomatoes treated with rhizobacteria showed increased resistance to the cucumber beetle (*Diabrotica undecimpunctata howardi* Barber) (Zehnder et al., 2003). These examples show that the soil microbial community and soil arthropods can have a strong influence on insect–plant interactions.

### 4.4 The impacts of climate on symbiosis

Changes in the abiotic environment can result in changes in the structure and function of insect‐associated microbiomes, and again this has the potential to impact other trophic levels. For example, increased temperatures have been associated with reduced *Wolbachia*‐mediated pathogen protection, while reduced temperatures have been associated with reduced *Spiroplasma*‐mediated parasitoid wasp defense (Corbin et al., 2021; Ross et al., 2017). In some systems, there are seasonal fluctuations in microbiome abundance or diversity that correlate with environmental temperatures and changes in microbiome functioning (Pintureau et al., 2002; Six & Bentz, 2007; Sumi et al., 2017). For instance, the gut microbial community of field crickets (*Gryllus veletis* Alexander & Gigelow) varies depending on seasonal conditions (Ferguson et al., 2018). This change in microbiome composition is correlated with decreased cricket survival during the winter when they are exposed to fungal infections (Ferguson et al., 2018). With increased weather extremes due to climate change, crickets may experience changes in mortality due to fungal infections, ultimately resulting in a shift in herbivore pressures and resource availability for their predators. Similarly, mosquitoes (*Anopheles coluzzii* Coetzee & Wilkerson), a vector of the malaria parasite (*Plasmodium* sp.), experience seasonal variation in microbiome composition that could affect mosquito survival during drought conditions (Krajacich et al., 2018), and reduced mosquito prevalence could ultimately reduce pathogen transmission rates. Interestingly, a recent study found that mosquitos in Africa ride high‐altitude winds to migrate to new locations (Atieli et al., 2023), which could facilitate pathogen transmission or expose mosquitoes to new microbial associates. Therefore, climate change presents an interesting challenge for assessing how this may alter the phenotypes of arthropods that environmentally acquire microbiomes.

The relationship between animals, plants, microbiota, and climate change has received considerable attention because of the potential impacts of these factors on the adaptive potential of insect species (Cavicchioli et al., 2019; Corbin et al., 2017; Renoz et al., 2019; Vitasse et al., 2021). Variations in temperature, CO_2_ levels, and/or plant phenology can affect insect distributions and associated microbial communities. For instance, alfalfa root nodulation is induced by *Rhizobium* bacteria, levels of which increased at elevated CO_2_ with 26°C temperatures (Ryalls et al., 2017). Subsequently, the lucerne weevil (*Sitona discoideus* Gyllenhal), an herbivore of alfalfa, also increased in emergence from these nodules (Ryalls et al., 2013). However, under elevated CO_2_ at 30°C, both root nodulation and weevil emergence decreased (Ryalls et al., 2013). In contrast, range expansion of potential insect pests is often facilitated by climate change. For example, some invasive aphids are better able to tolerate heat stress, due to genetic variation in the *ibpA* promoter region of their obligate symbiont, *B. aphidicola* (Burke et al., 2010; Dunbar et al., 2007). The temperature‐resistant variant of *B. aphidicola* might facilitate aphid expansion into warmer regions, which could further negatively impact plant health, alter the composition of parasitoid communities, and affect the abundance of ants that might use aphid honeydew secretions as a carbohydrate resource. Similarly, mountain pine beetles are becoming more common in northern regions due to shorter winters, which promote more optimal thermal conditions for insects and their symbionts (Robbins et al., 2022; Six & Bentz, 2007). The impacts of climate change on insect range expansion also scale up to the forest community, as increased numbers of pine beetles result in greater pine tree die‐offs, which subsequently impacts nutrient cycling in the ecosystem.

The trends discussed above often suggest paradoxical situations. Environmental disruption of insect–microbiota interactions in native species may lead to their decline; although in some instances the environmental range of the insect rather than microbiota may result in organism decline (Hammer et al., 2021). In contrast, potential pest arthropods and associated symbionts may thrive in new climatic conditions, fostering range expansion and biological invasions, and further declines in native species due to a variety of factors (e.g., competition, pathogen transmission, and habitat alteration). Thus, symbionts can benefit their arthropod hosts (i.e., ecologically facilitate) while causing detrimental impacts to surrounding organisms and the environment.

## 5 TARGETING AND INFLUENCING INSECT–MICROBE–ENVIRONMENT INTERACTIONS

The majority of symbiosis‐mediated pest management strategies implemented to date have largely focused on changing insect vectorial capacity (Bassene et al., 2018; Mendiola et al., 2020; Rio et al., 2004). The most widespread and successful example is *Wolbachia*, where the establishment of *Wolbachia* infections in the yellow fever mosquito (*Aedes aegypti* L.) blocks the spread of human pathogens, including dengue and Zika (O'Neill et al., 2018; Utarini et al., 2021). This strategy has now been employed in a dozen countries worldwide (WorldMosquitoProgram, 2021). These successes and the abundance of symbioses that affect pathogen transmission suggest ample opportunities for management strategies. *Wolbachia* confers viral resistance to several other insect hosts, and several ongoing efforts have focused on developing the application of these systems (Bourtzis, 2008; Brelsfoard & Dobson, 2009; Nikolouli et al., 2018). For instance, *Wolbachia* was recently established in the brown planthopper (*Nilaparvata lugens* Stål) to lower its vectorial capacity for the rice ragged stunt virus (Gong et al., 2020). Examining the potential of other microbial symbionts to block pathogen transmission may yield interesting discoveries. The presence of *Caballeronia* in squash bugs was found to significantly reduce transmission of the plant pathogen *Serratia marcescens*, the cause of cucurbit yellow vine disease (Mendiola et al., 2022), due to the competitive exclusion of the pathogen by *Cabelleronia*. Harnessing the presence of symbionts that stimulate immune priming or competitive exclusion, could be an effective control tactic for a variety of different insect pests in the future.

In contrast to pathogen‐blocking symbionts, other associations exacerbate the impact of pestiferous insects. Transferring symbiont strains from kudzu bugs (*Megacopta cribraria* Fabricius) that consume soybean (*Glycine max* (L.) Merr) to those that consume kudzu, showed that the symbiont *Candidatus* Ishikawaella capsulata was responsible for conferring pest status and the ability to consume soybeans (Brown et al., 2014; Hosokawa et al., 2008). Therefore, it may be beneficial to identify effective methods to prevent the acquisition of symbionts that enhance pest phenotypes.

In addition to impacts on vectorial capacity, microbial symbionts can affect other facets of insect biology, ultimately changing pest pressures. For example, when *A. aegypti* was infected with *Wolbachia*, this lowered the mosquito's thermal tolerance (Ware‐Gilmore et al., 2021), which could reduce the ability of this insect to extend its geographic range. In contrast, bird cherry‐oat aphids (*Rhopalosiphum padi* L.) infected with yellow barley virus (BYDV) received heat tolerance genes from the virus, making them more resistant to temperature increases (Porras et al., 2020), potentially increasing their pest potential for additional geographic regions. Therefore, it is essential to characterize the microbial symbiont composition of arthropod pests, understand how these symbionts function, and discover strategies for promoting or disrupting symbiosis.

An alternative to managing the resident symbiotic associations of beneficial and pestiferous insects is the use of paratransgenesis, which consists of the genetic modification of symbionts to alter host biology. An early example of this was the engineering of *Rhodococcus rhodnii*, a gut symbiont of the kissing bug (*Rhodnius prolixus* Stål), a vector of the Chagas disease parasite (*Trypanosoma cruzi* Chagas) (Beard et al., 1992). Paratransgenesis of *Rhodococcus rhodnii* results in the expression of an anti‐trypanosomal protein, reducing Chagas infection (Durvasula et al., 1999). For agricultural applications, *Pantoea agglomerans*, a gut symbiont of the glassy‐winged sharpshooter (*Homalodisca vitripennis* Germar), was engineered to express two antimicrobial peptides: melittin and a scorpine‐like molecule (Arora et al., 2018). These antimicrobial peptides are active against *Xylella fastidiosa*, the causative agent of Pierce's disease in grapes, ultimately disrupting pathogen transmission from insects to grapevines (Arora et al., 2018). Another application of paratransgenesis is the improvement of pollinator health by increasing pathogen resistance. *Snodgrassella alvi*, a symbiotic bacterium of the bee gut (*Apis mellifera* L.), has been engineered to produce dsRNAs that suppress deformed wing virus infection and reduce the survival of *Varroa* mites (Leonard et al., 2020). These examples highlight the potential for harnessing microbes to control arthropod pests and pathogens.

Increasing studies on insect‐associated microorganisms, microbial characterization, identification of functions, and ecosystem impacts, along with advances in molecular techniques, have created new opportunities for sustainable interventions aimed at improving or preventing the effects of insect pests. In agroecosystems, a widely used approach has focused on the application (i.e., biostimulants) or enhancement (i.e., biofertilizers) of microorganisms to improve plant growth or to control pathogens and pests (Daniel et al., 2022; Mitter et al., 2021; Rouphael & Colla, 2020). For example, inoculation with *Rhizobium* spp. to enhance the fertility of nutrient‐deficient soil (Fahde et al., 2023; Nobbe & Hiltner, 1896), the application of *Azospirillum* spp., a nitrogen‐fixing bacteria, to increase seed yield and aboveground biomass of wheat (Veresoglou & Menexes, 2010), and the use of arbuscular mycorrhizal fungi to increase the grain yield of many cereal crops (Zhang et al., 2019). Alternatives to this practice include endophytic colonization, where microorganisms residing inside a plant have pathogenic effects on insects (Gange et al., 2019; Hartley & Gange, 2009; Rivero et al., 2021). Strategies, such as using pollinators to disseminate biological control strains or endophytic seed colonizers to target crop flowers, called entomovectoring, have also shown promise (Smagghe et al., 2012). Exploiting competition, such as the use of yeast (*Aureobasidium pullulans*) and bacterial isolates (*Pantoea agglomerans*) that colonize flowers, prevented the establishment of the pathogen *Erwinia amylovora*, the causal agent of fire blight, and successfully controlled the disease in apple orchards (Pusey & Wend, 2012). As our knowledge of the different context‐dependent functions of microorganisms both singularly and in communities grows, we need to expand our catalog of beneficial and pathogenic microbes and microbial communities.

In addition to harnessing the benefits of individual microbes, manipulation of microbial communities provides additional avenues for discovery. The combination of synthetic biology, systems biology, microbial ecology, and experimental evolution has led to the engineering and construction of synthetic microbial communities (Großkopf & Soyer, 2014; Johns et al., 2016; Qian et al., 2020; Song et al., 2014). The use of microbial communities better reflects natural environments with complex interactions between species. These model systems are important for understanding the key ecological, structural, and functional features of communities in a controlled environment (Netzker et al., 2018). Additionally, synthetic microbial co‐cultivation has been increasingly explored for novel applications in biotechnology (Großkopf & Soyer, 2014). The generation and exploration of microbial communities provide opportunities to discover the chemicals they produce (Czajkowski et al., 2020; Netzker et al., 2018; Wang et al., 2023), which may aid in the management of pests and their pathogens, or enhance environmental remediation. The potential application could include promotion of plant growth (Morella et al., 2019; Quiñones‐Aguilar et al., 2016; Zhuang et al., 2021), increased disease resistance (Du et al., 2021), tackling pesticide degradation (Ahmad et al., 2022), and facilitating environmental bioremediation (Bhatt et al., 2021; Jaiswal & Shukla, 2020). Interestingly in a few instances, insect gut microbiota, particularly of Coleoptera, are reported to degrade plastic (Jang & Kikuchi, 2020). Plastics are an increasingly prolific waste product in the environment that could provide a food resource for some insects and entoremediation can break down larger plastics (Bulak et al., 2021), although the generation of microplastics remains a concern. There are many opportunities to harness resident and environmental microbes, in addition to generating synthetic microbial communities, and our next steps should include assessing the potential synergistic effects of different microbial community compositions.

There is an increased pace of insect biodiversity changes due to climate change, habitat modification, and chemical inputs to the surrounding environment (Lewinsohn et al., 2022; Outhwaite et al., 2022). Microbial symbionts may be the key to bolstering beneficial arthropod resilience and population persistence. Much like microbial symbionts that have been reported to enhance stress tolerance in pest insects, further investigation into the horizontal transfer of microbes to pollinators or natural enemies and their potential benefits is required. Akin to the supplementation of human diets with pre‐ or probiotics (e.g., fermented foods, dietary fiber, and/or microbial supplements), we should evaluate what combination of microbes might promote, for instance, increased pollinator health. Although distribution to large insect populations is likely to be unwieldy, a widespread mechanism of horizontal transmission could work, such as inoculating plant seeds that then transmit microbes to arthropods or spraying microbes on surfaces where arthropods can acquire them. Dispensing probiotic mixtures in food resources for animal husbandry operations may provide another tool to bolster beneficial arthropod populations and promote sustainable agriculture. For example, probiotics are used in insect mass‐rearing operations (Savio et al., 2022) and have the potential to be scaled to natural populations.

## 6 CONCLUSIONS

A continued focus on arthropod–microbe–environment interactions would greatly enhance the development of pest control practices by addressing the complexities that characterize most of our current pest challenges. While we are beginning to understand the roles that microbes play in a few select organisms, there are many opportunities to understand to what degree microbes or microbial communities can be used to manage pests and promote ecosystem health. In the instance of native and beneficial arthropods, inoculating or modifying microbial symbionts in arthropods or associated hosts may allow for more resilient organisms in the face of ongoing climate change.

Identifying additional ways to alter or enhance microbial composition can provide more sustainable approaches for the management of arthropod pests. This could generate increased resilience of agricultural crops or reduced pathogen transmission to animals, both of which are anticipated to positively impact organism health. For instance, there are ways in which we can harness existing beneficial microbes or develop synthetic microbial communities to bolster plant or animal health and reduce pest damage. Harnessing endemic microbes may enhance the long‐term success of remediation efforts, such as in areas with invasive plants, land with previous agricultural use, or former industrial and mining production sites. Conventional agricultural areas may experience depleted soils, which may benefit from inoculation with a commercial or synthetically assembled microbial community. Understanding the range of ecological niches of microbial organisms and how this may change insect–microbe associations may provide a key avenue to enhance pest management practices.

In the face of climate change, knowledge of environment‐by‐microbe and environment‐by‐organism‐by‐genotype interactions is essential. Understanding how microbes influence the recruitment or retention of other microbes in a system could provide effective approaches for promoting plant and animal health and reducing the negative impacts of insect pests or pathogens. Ultimately, we should continue to approach pest and climate change challenges from an innovative perspective that can aid in promoting sustainable and resilient ecosystems.

## AUTHOR CONTRIBUTIONS

**Jocelyn R. Holt:** Conceptualization (lead); visualization (lead); writing – original draft (lead); writing – review and editing (lead). **Nathalia Cavichiolli de Oliveira:** Conceptualization (supporting); writing – original draft (supporting); writing – review and editing (supporting). **Raul F. Medina:** Conceptualization (lead); writing – original draft (supporting); writing – review and editing (supporting). **Antonino Malacrinò:** Conceptualization (lead); visualization (supporting); writing – original draft (supporting); writing – review and editing (supporting). **Amelia R. I. Lindsey:** Conceptualization (supporting); visualization (supporting); writing – original draft (supporting); writing – review and editing (supporting).

## FUNDING INFORMATION

JRH was supported by an Agriculture and Food Research Initiative (AFRI) Competitive Grant from the U.S. Department of Agriculture (USDA) National Insitute of Food and Agriculture (NIFA) project number TEX09837 and accession number 1023098 during the writing of this manuscript. RFM was supported in part by the U.S. Department of Agriculture Hatch Program: TEX09185. ARIL was supported by the National Institute of General Medical Sciences (NIGMS) of the National Institutes of Health (NIH) under award number R35GM150991 and the National Institute of Allergy and Infectious Disease (NIAID) of the National Institutes of Health under award number R21AI175957.

## CONFLICT OF INTEREST STATEMENT

The authors declare no conflicts of interest.

## Data Availability

This is a review paper. Data sharing is not applicable to this article, as no datasets were generated or analyzed.
